# Aggressive dereplication using UHPLC–DAD–QTOF: screening extracts for up to 3000 fungal secondary metabolites

**DOI:** 10.1007/s00216-013-7582-x

**Published:** 2014-01-18

**Authors:** Andreas Klitgaard, Anita Iversen, Mikael R. Andersen, Thomas O. Larsen, Jens Christian Frisvad, Kristian Fog Nielsen

**Affiliations:** 1 Department of Systems Biology, Søltofts Plads, Technical University of Denmark, 2800 Kgs., Lyngby, Denmark; 2 Current address: Danish Emergency Management Agency, Universitetsparken 2, 2100 Copenhagen, Denmark

**Keywords:** Metabolomics, Mycotoxin, NRPS, LC–MS, UPLC, Polyketide, Nonribosomal peptide

## Abstract

In natural-product drug discovery, finding new compounds is the main task, and thus fast dereplication of known compounds is essential. This is usually performed by manual liquid chromatography-ultraviolet (LC-UV) or visible light-mass spectroscopy (Vis-MS) interpretation of detected peaks, often assisted by automated identification of previously identified compounds. We used a 15 min high-performance liquid chromatography–diode array detection (UHPLC–DAD)–high-resolution MS method (electrospray ionization (ESI)^+^ or ESI^−^), followed by 10–60 s of automated data analysis for up to 3000 relevant elemental compositions. By overlaying automatically generated extracted-ion chromatograms from detected compounds on the base peak chromatogram, all major potentially novel peaks could be visualized. Peaks corresponding to compounds available as reference standards, previously identified compounds, and major contaminants from solvents, media, filters etc. were labeled to differentiate these from compounds only identified by elemental composition. This enabled fast manual evaluation of both known peaks and potential novel-compound peaks, by manual verification of: the adduct pattern, UV–Vis, retention time compared with log D, co-identified biosynthetic related compounds, and elution order. System performance, including adduct patterns, in-source fragmentation, and ion-cooler bias, was investigated on reference standards, and the overall method was used on extracts of *Aspergillus carbonarius* and *Penicillium melanoconidium*, revealing new nitrogen-containing biomarkers for both species.

**Electronic supplementary material:**

The online version of this article (doi:10.1007/s00216-013-7582-x) contains supplementary material, which is available to authorized users.

## Introduction

Fungi are an immense source of diverse natural products that can be used as drugs, food and feed additives, and industrial chemicals [1, 2]. Unfortunately fungi also have a negative side, producing mycotoxins which include some of the most immunotoxic, estrogenic, cytotoxic, and carcinogenic compounds known [3, 4].

Fast and accurate dereplication of previously described compounds is an essential and resource-saving aspect of working with natural products [1, 5–9]. The alternative, isolation and subsequent NMR-based structure elucidation, is time consuming and costly [7], and is thus primarily used in important cases, e.g. for compounds with known bioactivity.

Currently, dereplication is mainly performed by liquid chromatography–mass spectrometry (LC–MS) analysis of extracts, followed by a search of all ions of interest performed by entering the monoisotopic mass into appropriate databases. For microbial compounds, the most comprehensive database is AntiBase (Wiley-VCH, Weinheim, Germany) the 2012 version of which contains 41,000 recorded compounds. In dereplication, obtaining an elemental composition is the most efficient first step because it reduces the number of hits from a database search 3–10-fold compared with searching for a nominal mass [9–11]. For compounds below 400–600 Da, high-resolution MS (HRMS) instruments can often provide the elemental composition unambiguously if they have < 0.5–1.5 ppm mass accuracy. In addition, time of flight (TOF)-based mass spectrometers can now provide an accurate isotope pattern, enabling an even higher degree of certainty for identification of elemental compositions [9, 12, 13].

An important extra detector is the UV–Vis diode array detection (DAD) detector, which provides information on the conjugated double-bond systems found in most secondary metabolites. This can be used to confirm or reject candidates from a database search [14, 15]. Finally, log D-based calculations can be used to predict the chromatographic elution order of compounds of interest [9].

Dereplication of peaks in extracts from genera, including *Aspergillus*, *Penicillium*, and *Fusarium*, which are known to produce many different compounds often results in many hits (1724, 1726, and 611 compounds, respectively, listed in AntiBase). Because of this, identifying compounds on the basis of UV–Vis, chromatographic retention, elution order, and comparison to biosynthetically related compounds is a slow (0.5–3 h per extract) and tedious task.

A solution could be to use MS–MS libraries [16] to identify compounds automatically. This is the preferred strategy in forensic science and toxicology, for which subjects commercial compound libraries are available [17]. However, no natural-product MS–MS libraries are currently available, because including an MS–MS spectrum for future dereplication is unfortunately not a prerequisite for publishing new structures. Because of this, only a few percent of described compounds from fungi are commercially available, and therefore only small in-house databases are available [9, 18, 19].

Another complication is that the compound adduct pattern and possible fragmentations need to be correctly interpreted, because unnoticed loss of water or addition of sodium or ammonium ions will invalidate a subsequent database search. Unambiguous determination of the accurate mass of fungal metabolites on the basis of adduct formation, dimers, and mutably charged ions can be challenging [9], but software including ACDs intelliXtract [19] and some instrument vendor software packages have algorithms for this.

To reduce the analysis time for known fungal compounds in complex extracts, we decided to test the TargetAnalysis software from Bruker Daltonics (similar software available from Waters, Thermo, Agilent, and Advanced Chemical Developments). The program was originally developed for pesticide [20] and forensic analysis [21]. TargetAnalysis can screen an extract for 3000 compounds, on the basis of mass accuracy, isotope fit, and retention time (RT), within 10–60 s, depending on how small peaks are integrated. The screening software was interfaced with our internal compound database, containing approximately 7100 compounds [9], via an in-house-built Excel application that generated automatic search lists for TargetAnalysis, and made it possible to search for the most likely adduct and/or fragment ions and to only include taxonomically relevant compounds if wanted.

Using this approach, we are able to rapidly screen extracts from several different fungi, and to annotate chromatographic peaks corresponding to known compounds. The approach makes it possible to easily identify chromatographic peaks that do not correspond to known compounds, thereby enabling one to quickly ascertain which compounds might be novel.

## Materials and methods

### Chemicals

Solvents were LC–MS grade, and all other chemicals were analytical grade. All were from Sigma-Aldrich (Steinheim, Germany) unless otherwise stated. Water was purified using a Milli-Q system (Millipore, Bedford, MA). ESI–TOF tune mix was purchased from Agilent Technologies (Torrance, CA, USA).

Reference standards of mycotoxins and microbial metabolites (approximately 1500, 95 % of fungal origin) had been collected over the last 30 years [9, 22, 23], either from commercial sources, as gifts from other research groups, or from our own projects. Approximately one-third of the standards were purchased from Sigma-Aldrich, Axxora (Bingham, UK), Cayman (Ann Arbor, MI), TebuBio (Le-Perray-en-Yvelines, France), Biopure (Tulln, Austria), Calbiochem, (San Diego, CA), and ICN (Irvine, CA). Standards were maintained dry at −20 °C, and were compared with original UV–VIS data, accurate mass, and relative RT from previous studies [22].

Culture extracts in the examples originated from three-point cultures on solid media, incubated for seven days in darkness at 25 °C, and extracted using a (3:2:1) (ethyl acetate:dichloromethane:methanol) mixture [24]. *Penicillium melanoconidium* IBT 30549 (IBT culture collection, author’s address) was grown on CYA, and *A. carbonarius* IBT 31236 (ITEM5010) was grown on YES [24].

### UHPLC–DAD–QTOFMS

A UHPCL–DAD–QTOF method was set up for screening, with typical injection volumes of 0.1–2 μl extract. Separation was performed on a Dionex Ultimate 3000 UHPLC system (Thermo Scientific, Dionex, Sunnyvale, California, USA) equipped with a 100 × 2.1 mm, 2.6 μm, Kinetex C_18_ column, held at a temperature of 40 °C, and using a linear gradient system composed of A: 20 mmol L^−1^ formic acid in water, and B: 20 mmol L^−1^ formic acid in acetonitrile. The flow was 0.4 ml min^−1^, 90 % A graduating to 100 % B in 10 min, 100 % B 10–13 min, and 90 % A 13.1–15 min.

Time-of-flight detection was performed using a maXis 3G QTOF orthogonal mass spectrometer (Bruker Daltonics, Bremen, Germany) operated at a resolving power of ~50000 full width at half maximum (FWHM). The instrument was equipped with an orthogonal electrospray ionization source, and mass spectra were recorded in the range *m*/*z* 100–1000 as centroid spectra, with five scans per second. For calibration, 1 μl 10 mmol L^−1^ sodium formate was injected at the beginning of each chromatographic run, using the divert valve (0.3–0.4 min). Data files were calibrated post-run on the average spectrum from this time segment, using the Bruker HPC (high-precision calibration) algorithm.

For ESI^+^ the capillary voltage was maintained at 4200 V, the gas flow to the nebulizer was set to 2.4 bar, the drying temperature was 220 °C, and the drying gas flow was 12.0 L min^−1^. Transfer optics (ion-funnel energies, quadrupole energy) were tuned on HT-2 toxin to minimize fragmentation. For ESI^−^ the settings were the same, except that the capillary voltage was maintained at −2500 V. Unless otherwise stated, ion-cooler settings were: transfer time 50 μs, radio frequency (RF) 55 V peak-to-peak (Vpp), and pre-pulse storage time 5 μs. After changing the polarity, the mass spectrometer needed to equilibrate the power supply temperature for 1 h to provide stable mass accuracy.

### Construction of the compound database

The database was constructed in ACD Chemfolder (Advanced Chemistry Development, Toronto, Canada) from:

1. reference standards (~1500) [9];
2. tentatively identified compounds (~500) [25–27];
3. compound peaks appearing in blank samples; and
4. all compounds in AntiBase2012 listed as coming from: *Aspergillus*, *Fusarium*, *Trichoderma*, *Penicillium*, *Chaetomium*, *Stachybotrys*, *Alternaria*, and *Cladosporium*.

A detailed description of the database construction can be found in the Electronic Supplementary Material, Section “Introduction”.

For each compound, the known or suspected major adducts were registered as: [M + H]^+^, [M + Na]^+^, [M + NH_4_]^+^, [M + K]^+^, [M + H + CH_3_CN]^+^, [M + Na + CH_3_CN]^+^, [M + H − H_2_O]^+^, [M + H − 2H_2_O]^+^, [M + H − H_2_]^+^ (sterols), [M + H − HCOOH]^+^, [M + H − CH_3_COOH]^+^, [M + 2H]^2+^, [M + Na + H]^2+^ or [M + 2Na]^2+^ or “No ionization” in ESI^+^, and in ESI^−^: [M − H]^−^, [M − H + HCOOH]^−^, and [M + Cl]^−^.

### Creating search lists for targetanalysis

A Microsoft Excel application was created for sorting the Chemfolder database into a taxonomically relevant search-list for TargetAnalysis (elemental composition and charge state of desired adduct, and name of compound).

For labeling peaks in Bruker DataAnalysis 4.0 (DA), compounds that were available as reference standards were labeled “S-x” in front of the name. A description of the database creation procedure can be found in the Electronic Supplementary Material, Section “Introduction”.

### Automated screening of fungal samples

TargetAnalysis 1.2 (Bruker Daltonics, Bremen, Germany), was used to process data-files, with the following typical settings:

A) retention time (if known) as ± 1.2 min as broad, 0.8 min as medium, and 0.3 min as narrow range;
B) SigmaFit; 1000 (broad) (isotope fit not used), 40 (medium), and 20 (narrow); and
C) mass accuracy of the peak assessed at 4 ppm (broad), 2.5 ppm (medium), and 1.5 ppm (narrow).

Area cut-off was set to 3000 counts as default, but was often adjusted for very concentrated or dilute samples.

The software DataAnalysis (DA) from Bruker Daltonics was used for manual comparison of all extracted-ion chromatograms (EIC) generated by TargetAnalysis to the base peak chromatograms (BPC), to identify non-detected major peaks.

## Results and discussion

### The database

The database used for screening comprised 7100 compounds, of which 1500 were available reference standards and 500 were tentatively identified compounds. The database was handled in ACD Chemfolder, using a custom interface shown in Fig. S1, Electronic Supplementary Material. The database also contained legacy data from older HPLC–DAD [22], HPLC–DAD–TOFMS [9, 23], and pKa data [9] if available. Records from AntiBase needed proofreading, because we found that approximately 2–3 % of the structures had incorrect elemental compositions. We also estimate that approximately 5 % of structures published annually are not indexed.

**Fig. 1:**
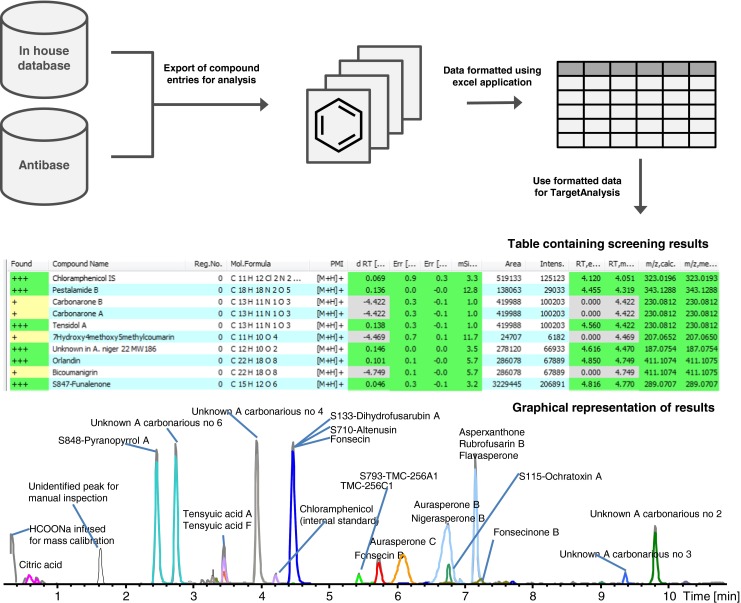
Example of workflow for screening of fungal extracts, in this case an extract from *Aspergillus niger*. The database maintained at our center contains 7100 records, comprising reference standards and their associated MS and UV data. For a specific analysis it is possible to export relevant entries from the database and, via an in-house-built Excel application, convert these to a format that can be imported into TargetAnalysis. Analysis via TargetAnalysis then yields both a graphical interpretation of the results and a table of the data

Because TargetAnalysis could not extract both targeted and untargeted data and combine them, the fastest workflow was to overlay all the identified compounds from TargetAnalysis on the BPC chromatograms. All major non-identified peaks could then easily be observed visually (as shown in Fig. 1), dereplicated, and added to the database as a tentatively identified [9, 25] or unknown compound. Subsequently it was clear that the signals from compounds originating from filters, media blanks etc. were most efficiently handled by including them in the database, so that they would be annotated and labeled by TargetAnalysis. This led to labeling peaks with the reference standard number (Fig. 1), indicating whether a compound was available as a reference standard for subsequent reanalysis.

The results from the analysis of an extract from *A. niger* are depicted in Fig. 1, illustrating the major disadvantage of the method. It can be seen that several compounds have been annotated to the same chromatographic peak, because numerous compounds in the search list had the same elemental composition and unknown RT. This is the major reason for not including, e.g., all 41,000 compounds from AntiBase2012 in the search list, because it contains up to 130 compounds with the same elemental composition [9]. For each experiment it is therefore important to use a search list from which highly unlikely compounds, for example metabolites from other organisms, are restricted. If no compounds are found, reanalysis can be conducted using a list of all elemental compositions in the database of choice.

### Handling adducts and in-source fragmentation

Early analytical work (results not shown), using atmospheric-pressure chemical ionization (APCI)^+^, APCI^−^, ESI^+^ and ESI^−^ ionization for analysis of extracts from *A. niger* and *A. nidulans*, did not reveal superior ionization by APCI over ESI for any compound. Thus APCI was not further pursued, although there must be some apolar and/or semi-volatile compounds that are better ionized by APCI.

Adduct formation on the maXis 3G ion-source was surprisingly different from that observed on our 10-years-older Waters Micromass LCT (z-spray source) [9], even though exactly the same eluents were used. In ESI^+^ mode we observed many compounds using the maXis, e.g. chloramphenicol and several anthraquinones, which were not previously detected by the LCT system using ESI^+^. It remains to be investigated whether this was caused by the grounded needle (and thus a potential of −42000 V over the source), the ion-funnel, or other changes in the source. Ammonium adducts were also far less abundant on the maXis, and formation seemed to be efficiently suppressed by the drying gas, leading to spectra with abundant [M + H]^+^ and [M + Na]^+^, because most compounds with high affinity for ammonium also have a high affinity for sodium [9].

An interesting phenomenon observed with ESI^+^ was that in the end of the gradient, when the acetonitrile content was close to 100 %, ionization seemed to favor formation of [2M + Na]^+^ ions. For such analytes as the variecoxanthones and emericellin (Fig. S2, Electronic Supplementary Material) the [2M + Na]^+^ ion (*m*/*z* 839.3766) had a 5–10-fold-higher intensity than [M + H]^+^. This was presumably caused by the high acetonitrile content, which would have facilitated fast evaporation, and acidic compounds may thus hold the residual Na^+^ by ion exchange before evaporation from the droplet.

Macrocyclic trichothecenes in extracts from *Baccharis megapotamica* [28] revealed that the adduct pattern was concentration-dependent, with the highest intensity [M + Na]^+^ occurring at low concentrations of the analyte (Fig. S3, Electronic Supplementary Material). This is probably the result of limited Na^+^, and thus [M + H]^+^ is most abundant when Na^+^ is depleted. On full-scan instruments this phenomenon can be regarded as *adduct displacement*, whereas it will be observed as ion suppression on MS–MS instruments if only one of [M + H]^+^ or [M + Na]^+^ is measured. For MS–MS characterization of compounds that favor sodium adducts, we have in several applications used ammonium formate as buffer to depress sodium adduct formation. In one example we also changed the sodium formate calibration solution to a polyethylene glycol mixture, and switched the glass water-solvent bottle to plastic.

Ergosterol and related steroles were, surprisingly, detected as [M + H − H_2_]^+^ ions, whereas, e.g., cholesterol was detected as [M + H − H_2_O]^+^.

ESI^−^ ionized acidic compounds (carboxylic acids, enoles and phenols) well, because of easy disassociation of H^+^, and also proved superior to ESI^+^ unless the target compounds also contained amine or amide functionalities. Compounds without acidic protons, that were observed as [M + HCOO]^−^ on both Waters LCT z-spray source instrumentation [9] and an Agilent 6550 QTOF, were often not detected at all using the maXis system.

Ion-source fragmentation was unavoidable for very fragile molecules, but was mainly observed as water loss for compounds that formed sodium adducts: jumping from [M + Na]^+^ to [M + H − H_2_O]^+^, with *m*/*z* 39.9925, and occasionally also to [M + H − 2H_2_O]^+^, with *m*/*z* 58.0031. Thus the sodium adducts could be an advantage when screening fragile compounds. Cases where [M + H]^+^ was not observed were much more predominant on the maXis than on the Waters LCT (z-spray source). In-source fragmentation could be minimized by lowering the potential of the quadrupole and between the funnels, but could not be abolished because this would lead to >10 % loss of sensitivity. We therefore included [M + H − H_2_O]^+^ and [M + H − 2H_2_O]^+^ in the database of compounds losing H_2_O during ESI^+^ (often an alcohol group with α-carbon was available for elimination via double-bond formation) [9].

The screening process was also performed, using similar samples, on an Agilent 1290 UHPLC–6550 QTOF system, using Agilent Masshunter’s *Find By Formula* option. This function could handle different adducts and simple losses, for example water loss, theoretically ensuring that no compounds were overlooked. This, however, also resulted in many more false positives, because all peaks are believed to correspond to, e.g., an [M + H − H_2_O]^+^ ion, even if the peaks also fit the [M + H]^+^ of another compound. ACD’s MS Workbook Suite intelliXtract function (v. 12) was also tested. The software could assign the whole adduct, multimer and fragment pattern for a peak, but required the presence of a [M + H]^+^ or [M − H]^−^ ion. This software was approximately 50–100 times more time-consuming than Brukers TargetAnalysis for a list of 3000 compounds, but does work for smaller databases [19].

Molecules with masses above 1000 Da, which include many NRPs (e.g. lipopeptides and peptaibols), all produced doubly and often also triply charged ions, thus appearing in the scan window of *m*/*z* 100–1000. The only two exceptions were special cyclic peptides, for example cereulide and valinomycin, which are very strong K^+^-ionophores and therefore only produced [M + Na]^+^ and [M + K]^+^ ions [29].

**Fig. 2:**
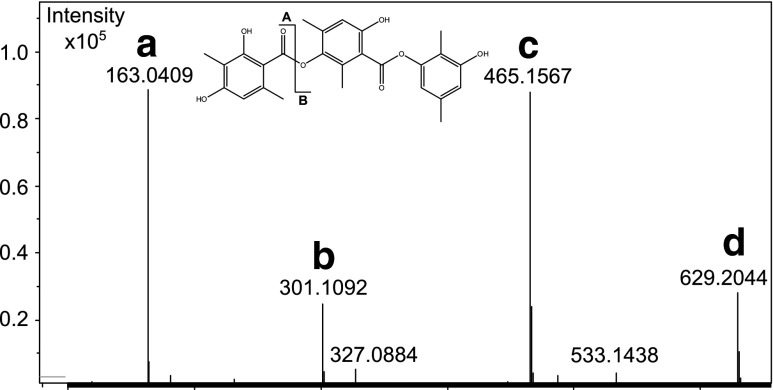
ESI^−^ spectrum of 3-Hydroxy-2,5-dimethylphenyl 3-[(2,4-Dihydroxy-3,6-dimethylbenzoyl)oxy]-6-hydroxy-2,4-dimethylbenzoate, showing M − H]^−^ (C) and fragment ions **a** and **b**. **d** is a composite of ions **a** and **c**

The adduct formation behavior of some compounds can however be hard to predict. This was observed for an extract of *Phoma levellei* [30] (incorrectly identified as *Cladosporium uredinicola*), for which the ESI^−^ spectrum of 3-Hydroxy-2,5-dimethylphenyl 3-[(2,4-Dihydroxy-3,6-dimethylbenzoyl)oxy]-6-hydroxy-2,4-dimethylbenzoate (Fig. 2) indicated the presence of several co-eluting compounds. Deconvolution of the ions revealed that ions labeled A–D came from the same compound. Ion C corresponded to [M − H]^−^, A and B were fragments, and D was a composite ion of [M − H]^−^ and one fragment-ion A.

### Ion-cooler bias

The maXis 3G is equipped with a hexapole ion-cooler, which collects the ions, reduces their kinetic energy, and ejects them into the orthogonal accelerator in the TOF mass analyzer. Our results reveal that the ion cooler settings have a significant effect on the intensities of the ions in the measured mass range (Fig. S4, Electronic Supplementary Material).

Three variables were important:

1. the *ion*-*cooler radio frequency* (RF), which sets the voltage for the ion-cooler;
2. the transfer time, which is the time window wherein ions are transmitted into the TOF; and
3. the *pre*-*pulse storage time*, which will apply a low mass limit and is a delay between the transfer time and the TOF pulser. Higher values favored the transfer of higher *m*/*z* ions, but also discriminated low *m*/*z* ions.

Figure S4 (Electronic Supplementary Material) shows selected results from analysis using seven different transfer times. The results revealed that the ion-cooler “window” for low mass compounds is narrow, and the settings used to obtain an optimum signal for lower *m*/*z* ions resulted in low intensities of higher *m*/*z* ions, and vice versa. For analytes with *m*/*z* lower than 100 (data not shown), the optimum settings excessively discriminated the signal intensity of higher *m*/*z* values. At an ion cooler RF value of 30 Vpp, the signal of *m*/*z* 91 was highly suppressed at all transfer times.

Our in-house database contained 7100 compounds with a [M + H]^+^ in the range *m*/*z* 100–1000. Of these, 14 % will have a [M + H]^+^ < 226 *m*/*z* and will reach only 30 % of their maximum intensity using standard screening settings. For ions smaller than *m*/*z* 130 the signal suppression will be extensive, but luckily less than 1 % of the compounds in our in-house database and AntiBase have masses this low [9]. If a target compound was in the mass range below *m*/*z* 130, the optimum ion-cooler settings resulted in an intensity of less than 10 % for compounds with an *m*/*z* > 226, and of only 5 % of the signal from compounds with an *m*/*z* > 600. It is important to be aware of this signal discrimination in some mass ranges under different ion-cooler settings.

### Effect of detector overload on isotope pattern and mass accuracy

Because fungal extracts contain many different compounds with varying concentrations and ionization efficiencies, screening of extracts routinely resulted in analysis of compounds with intensities higher than 2–3 × 10^6^ counts, which overloaded the detector of the maXis QTOF (this problem was much more severe on older TOF instruments [9]). This caused an *m*/*z* shift to higher values, which in the worst case resulted in an increase of up to 3–4 ppm. This also led to a distorted isotopic pattern, where the A + 1, A + 2 isotopomers were too intense relative to the A isotopomer. To avoid false negative results in TargetAnalysis, it was thus crucial to set a wide range (5 ppm) on the isotope fit and mass accuracy. However, these high-intensity peaks could be easily spotted by the peak height in the results table, after which data for the chromatographic peak could be examined from scans where the detector was not overloaded. The isotope fit was highly dependent on a weekly detector tuning, and the medium and narrow-range settings had to be increased twofold when the detector had not been tuned within the week.

### Aggressive dereplication reveals new metabolites from highly toxic spoilage fungus *Aspergillus carbonarius*

*A. carbonarius* is a physiologically very well investigated species because of its contamination of grapes, and the subsequent contamination of wine and raisins, with ochratoxin A [31]. However, other compounds from the fungus have attracted little attention. As well as this toxin, it is capable of producing carbonarones and pestalamide A (former tensidol B) [32], pyranonigrins, carbonarins, organic acids, and aurasperones [26].

Extracts from *A. carbonarius* cultivated on YES agar were screened for 3000 compounds:

1. compounds from *Aspergillus* (with an emphasis on *Aspergillus* section Nigri compounds ) and *Penicillium*;
2. all standards available in our collection; and
3. all unidentified peaks registered in our database.

**Fig. 3:**
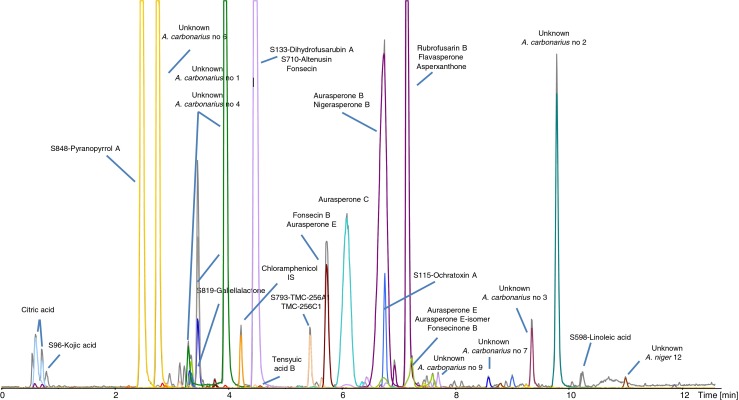
Analyzed fungal extract from *A. carbonarius* cultivated on YES media. The chromatogram is overlaid with EIC from detected compounds, facilitating easy dereplication. The chromatogram has been scaled to better illustrate the smaller peaks

With a high area cut-off of 10,000 counts, 66 peaks were integrated (Table 1); however, 16 of these compounds were from peaks assigned to several compounds (up to five) and thus only 45 true peaks were annotated. The major peaks in the sample are displayed in Fig. 3.

**Table 1:** Results from the aggressive dereplication of an extract of *Aspergillus carbonarius* grown on YES agar

Peak	Class	Comment	Compound name	Molecular formula	Err (ppm)	mSigma	Area (arbitrary units)	RT measured (min)	RT expected (min)
A	+++	OK double peak caused by injection	Citric acid	C_6_H_7_NaO_7_	0.1	8	351577	0.609	0.61
B	+++	OK double peak caused by injection	Citric acid	C_6_H_7_NaO_7_	0.1	3	256614	0.719	0.72
C	+++		BL-UK Cla no 60 pos. blank	C_10_H_13_N_5_O_4_	0.9	7	22958	0.722	0.72
D	+	Wrong, UV and RT do not fit	S96-Kojic acid	C_6_H_6_O_4_	0.9	9	14965	0.791	1.2
E	+++		BL-UK Cla no 72 pos. blank	C_10_H_16_N_2_O_2_	0.2	11	15379	1.807	1.75
F	+++		BL-UK Cla no 95 pos. blank	C_7_H_14_N_2_O_3_	1.2	6	15141	2.243	2.1
G	+++	OK	S848-Pyranonigrin A	C_10_H_9_N_1_O_5_	0.9	19	5428853	2.475	2.36
H	+++		UK in A. ni 2	C_10_H_9_N_1_O_4_	0.4	17	24641	2.756	2.906
I	+++	Interesting new biomarker	UK A car no 6	C_11_H_11_N_1_O_5_	0.6	17	5203919	2.756	2.751
J	+++		UK in A. ni 19	C_18_H_37_NaO_10_	0.2	10	13945	2.892	2.844
K	+++		BL-UK Cla no 11 pos. blank	C_11_H_18_N_2_O_2_	1.3	10	29484	2.912	3.09
L	+++		UK in A. ni 2	C_10_H_9_N_1_O_4_	1.2	1	90082	2.962	2.906
M	+++		BL-UK Cla no 12 pos. blank	C_11_H_18_N_2_O_2_	0.2	5	44764	3.14	3.09
N	+++	Interesting new biomarker	UK A car no 4	C_18_H_21_N_1_O_2_	0.1	16	350827	3.295	3.288
O	+++		UK in A. ni 16	C_22_H_45_NaO_12_	0.6	18	13611	3.299	3.25
P	+	No confused by the A isomer	Tensyuic acid A	C_11_H_16_O_6_	0.2	7	96858	3.344	0
P	+	Presumably OK	Tensyuic acid F	C_11_H_16_O_6_	0.2	7	96858	3.344	0
Q	++		UK A car no 4	C_18_H_21_N_1_O_2_	0.1	15	48785	3.592	3.288
Q	++		UK A car no 1	C_18_H_21_N_1_O_2_	0.1	15	48785	3.592	3.923
R	+++		UK in A. ni 5	C_21_H_44_O_11_	0.3	14	10039	3.63	3.581
S	+	OK but may be the C isomer	Pyranonigrin B	C_11_H_11_N_1_O_6_	0.5	9	55596	3.76	0
S	+	OK but may be the B isomer	Pyranonigrin C	C_11_H_11_N_1_O_6_	0.5	9	55596	3.76	0
T	+++		UK in A. ni 7	C_23_H_47_NaO_12_	0.4	37	17040	3.767	3.72
U	++		UK A car no 4	C_18_H_21_N_1_O_2_	0.7	15	5265217	3.944	3.288
U	+++		UK A car no 1	C_18_H_21_N_1_O_2_	0.7	15	5265217	3.944	3.923
V	+		Pyranonigrin D	C_11_H_9_N_1_O_5_	0.2	9	17070	3.946	0
W	+++	Internal standard	Chloramphenicol IS	C_11_H_12_Cl_2_N_2_O_5_	0.2	31	326301	4.219	4.12
X	+++	No confused by Fonsecin	S133-Dihydrofusarubin A	C_15_H_14_O_6_	1.1	25	6829770	4.47	4.75
X	++	Wrong, UV and RT do not fit	S710-Altenusin	C_15_H_14_O_6_	1.1	25	6829770	4.47	4.908
X	+++	OK	Fonsecin	C_15_H_14_O_6_	1.1	25	6829770	4.47	4.45
Y	+	OK but one must be a new isomer	Tensyuic acid B	C_12_H_18_O_6_	1.1	24	21361	4.554	0
Z	+	OK but one must be a new isomer	Tensyuic acid B	C_12_H_18_O_6_	1	22	10189	4.681	0
AA	+++	OK	S133-Dihydrofusarubin A	C_15_H_14_O_6_	1	46	10340	5.031	4.75
AA	+++	Wrong, UV and RT do not fit	S710-Altenusin	C_15_H_14_O_6_	1	46	10340	5.031	4.908
AB	++	No confused by Dihydrofusarubin A	Fonsecin	C_15_H_14_O_6_	1	46	10340	5.031	4.45
AC	++		Aurasperone C	C_31_H_28_O_12_	0.5	37	15414	5.249	5.94
AD	+++	No confused by TMC-256A1	TMC-256C1	C_15_H_12_O_5_	0.6	18	349791	5.437	5.67
AD	+++	OK	S793-TMC-256A1	C_15_H_12_O_5_	0.6	18	349791	5.437	5.37
AE	++		Aurasperone C	C_31_H_28_O_12_	0.4	41	19423	5.494	5.94
AF	+++	OK	TMC-256C1	C_15_H_12_O_5_	0.3	7	65429	5.641	5.67
AF	+++	No confused by TMC-256C1	S793-TMC-256A1	C_15_H_12_O_5_	0.3	7	65429	5.641	5.37
AG	+++		Fonsecin B	C_16_H_16_O_6_	0.8	30	1055089	5.729	5.66
AH	+	Wrong water-loss ion of C isomer	Niasperone C	C_31_H_26_O_11_	1	9	76397	6.08	0
AH	+++	Wrong water-loss ion of C isomer	Aurasperone F	C_31_H_26_O_11_	1	9	76397	6.08	6.303
AH	+++		Aurasperone C	C_31_H_28_O_12_	1.1	23	3247597	6.081	5.94
AI	++		UK in A. ni 23	C_15_H_33_N_17_O_6_	0.2	62	39935	6.344	6.23
AJ	++		UK in A. ni 20	C_28_H_36_N_4_O_5_	0.9	25	49747	6.397	6.043
AK	+	OK but may be a different isomer	Niasperone C	C_31_H_26_O_11_	0.8	11	115620	6.434	0
AK	+++	OK but may be a different isomer	Aurasperone F	C_31_H_26_O_11_	0.8	11	115620	6.434	6.303
AL	+++	Wrong water-loss ion of B isomer	Aurasperone E	C_32_H_28_O_11_	0.9	23	186091	6.728	6.62
AL	++	Wrong water loss ion of B isomer	Aurasperone E-isomer	C_32_H_28_O_11_	0.9	23	186091	6.728	7.104
AL	++	Wrong water loss ion of B isomer	Fonsecinone B	C_32_H_28_O_11_	0.9	23	186091	6.728	7.472
AL	+	OK but may be a different isomer	Niasperone B	C_32_H_30_O_12_	1.3	22	6659679	6.728	0
AL	+++	OK but may be a different isomer	Aurasperone B	C_32_H_30_O_12_	1.3	22	6659679	6.728	6.605
AM	+++	OK	S115-Ochratoxin A	C_20_H_18_Cl_1_N_1_O_6_	0.7	50	693721	6.75	6.62
AN	+	OK but may be a different isomer	Niasperone C	C_31_H_26_O_11_	1.5	9	62334	6.779	0
AN	++	OK but may be a different isomer	Aurasperone F	C_31_H_26_O_11_	1.5	9	62334	6.779	6.303
AO	++	No rubrofusarin	Flavasperone	C_16_H_14_O_5_	0.7	20	146028	6.923	7.2
AO	+++	OK	Rubrofusarin B	C_16_H_14_O_5_	0.7	20	146028	6.923	7.029
AP	+++	OK	Flavasperone	C_16_H_14_O_5_	0.6	14	4285585	7.145	7.2
AP	++	No flavasperone	Rubrofusarin B	C_16_H_14_O_5_	0.6	14	4285585	7.145	7.029
AQ	++	OK but may be a different isomer	Aurasperone E	C_32_H_28_O_11_	0.2	35	300587	7.221	6.62
AQ	+++	OK but may be a different isomer	Aurasperone E-isomer	C_32_H_28_O_11_	0.2	35	300587	7.221	7.104
AQ	+++	OK but may be a different isomer	Fonsecinone B	C_32_H_28_O_11_	0.2	35	300587	7.221	7.472
AR	+++	OK but may be a different isomer	Fonsecinone B	C_32_H_28_O_11_	0.7	15	156648	7.588	7.472
AS	+++		S598-Linoleic acid	C_18_H_32_O_2_	0.6	11	104992	10.23	10.17

mSigma, fit of isotope pattern (see text for more details); RT, retention time

Citric acid was detected as the sodium adduct and as two peaks because of poor retention on the column, which occurred because the LC–MS method is not well suited to such polar compounds. Kojic acid was incorrectly identified as another compound with the same elemental composition, because neither the RT nor the characteristic UV spectrum matched a reference standard.

Three interesting nitrogen-containing biomarkers for this species, with elemental compositions C_11_H_11_NO_5_ and C_18_H_21_NO_2_ (two isomers), were detected (unknown 1, 4, and 6), and these were not detected for other black *Aspergilli* (results not shown). Ochratoxin A, which was produced in very high amounts, is an interesting case because its precursors, ochratoxin α and B, were not detected even in trace amounts, indicating that the biosynthetic enzymes are very efficient.

Several closely eluting same-elemental-composition groups were observed and needed manual verification. For example, the rationale for identifying peak AA, as seen in Table 1, was:

1. Altenusin C_15_H_14_O_6_ was from *Alternaria* and thus taxonomically unlikely. RT was within the limits where a reference standard should be co-analyzed in the sequence for verification. Inspection of the UV–Vis data led to easy elimination, and so did the presence of a perfectly co-eluting [M + Na]^+^ ion with M = C_15_H_16_O_7_.
2. Fonsecin could be eliminated by the same arguments.
3. Finally, dihydrofusarubin A was identified as the correct compound, on the basis of its perfectly matching UV–Vis spectrum and its [M + H − H_2_O]^+^ and [M + Na]^+^ ions. However, dihydrofusarubin A was only detected because it was registered in the database in the form [M + H − H_2_O]^+^.

The AL peak (Table 1) must be niasperone B or aurasperone B, but could not be differentiated without a reference standard. In that case, water-loss ions led to the peak being wrongly assigned to aurasperone E and one of its isomers, and to fonsecinone B.

The pair flavasperone and rubrofusarin B should both be produced when the dimeric naphtho-γ-pyrones are produced, and a log D calculation revealed that rubrofusarin B should elute first.

Differentiating the tensyuic acids was more ambiguous, because the reported elution pattern from reversed phase is F, A, B, C, D, and E [33], with F and B having the same elemental composition, and A and B almost co-eluting. Manual inspection of the screening results was therefore necessary to attempt to distinguish between the isomers. This revealed that the first-eluting tensyuic acid was most probably the F isomer (1.3 min to the B isomer). However, the B isomer could not be unambiguously assigned as one of the two peaks Y or Z, because only one compound with C_12_H_18_O_6_ is described.

In conclusion, the method very quickly identified suspected compounds from *A. carbonarius*. Besides this, a novel group of nitrogen-containing compounds, and tensyuic acids and numerous other compounds from related species, were detected. This indicated that, from a toxicological perspective, more compounds needed to be considered. A problem is that many of the closely related niasperones, aurasperones, and fonsecinones have identical elemental compositions and UV–Vis spectra and are very difficult to differentiate. To enable differentiation, we are currently considering an MS–HRMS library approach, as done for a toxic substance library [17]. However, TargetAnalysis does not presently have the capability to handle MS–HRMS data or pseudo-MS–MS data including MS-E, MS-All and/or All-Ions [21]. A further example of aggressive dereplication applied to *Penicillium melanoconidium* can be found in Electronic Supplementary Material Section “Materials and methods” and Tables S1 and S2. Here, several families of compounds not previously seen in the species were detected (Fig. S5, Electronic Supplementary Material). This included the highly toxic verrucosidins, and a presumed novel dideoxyverrucosidin. Chrysogine, a compound often detected in cereal-infecting Fusaria, was also detected, indicating that this may be an important virulence factor. The example shows how the aggressive dereplication procedure was used to detect known compounds not previously detected from the fungus. The results illustrate that all major peaks in the chromatogram were overlaid with an EIC, proving the effectiveness of the procedure and also indicating that it is a chemically very well characterized species.

## Conclusion

Screening fungal secondary metabolites on the basis of elemental composition and lists restricted to the same genus and related fungi was proved to be an efficient way to quickly investigate fungal extracts. By overlaying detected peaks and BPC chromatograms, the approach gives a visual overview of a sample and indicates whether it is a previously uninvestigated species by establishing how many peaks are unlabeled. This approach can also be used on other vendor instrumentations using analogous software packages, for example: TargetLynx (Waters), TraceFinder (Thermo), MassHunter Find By Formula (Agilent), and ACD intelliXtract (Advanced Chemical Developments).

Labeling of co-identified biosynthetic related compounds could also be directly identified from the peak, making it possible to quickly assess the elution order of such compounds.

However, adduct formation and simple fragmentations are still important challenges to address when working with analytes that do not only form [M + H]^+^ or [M − H]^−^. Using a database approach and learning from the spectrometric behavior of reference standards can minimize problems with false-negative results. More efficient adduct-analysis software will further improve this setup [9, 21].

A further improvement to be introduced is use of MS–MS [17, 19, 34] and/or pseudo-MS–MS (MS-All, MS-E, All Ions) [21] to obtain compound-specific fragment ions for confirmation of reference standards, reducing the need to run many thousands of reference standards on a daily basis. The addition of qualifier and/or fragment ions from libraries and literature data will help to minimize the number of wrongly annotated ions with the same elemental composition, which is the main disadvantage of this method.

## Electronic supplementary material

Below is the link to the electronic supplementary material.

ESM 1 (PDF 1.00 MB)
