# Supplementary material for: Aggressive dereplication using UHPLC–DAD–QTOF: screening extracts for up to 3000 fungal secondary metabolites
Source: Anal Bioanal Chem. 2014 Jan 18;406(7):1933–43. doi: 10.1007/s00216-013-7582-x (PMC3955480; doi:10.1007/s00216-013-7582-x)
Supplement: Supplementary file 1 — (PDF 1.00 MB) [file 216_2013_7582_MOESM1_ESM.pdf]

Analytical and Bioanalytical Chemistry

Electronic Supplementary Material

Aggressive dereplication using UHPLC-DAD-QTOF: screening extracts for up to 3000 fungal secondary metabolites

Andreas Klitgaard, Anita Iversen, Mikael R. Andersen, Thomas O. Larsen, Jens Christian Frisvad, Kristian Fog Nielsen

Section 1. Construction of compound database

The database was constructed in ACD Chemfolder (Advanced Chemistry Development, Toronto, Canada) from: i) our in-house collection of reference standards (~1500 compounds) [1]; ii) compounds tentatively identified during the last 30 years (~500 compounds) [2-5]; iii) compound-peaks appearing in blank samples; iv) putative biosynthetic intermediates mainly from *A. niger* and *A. nidulans* and PKS pathways; and v) all compounds in AntiBase2012 which were listed as coming from: *Aspergillus, Fusarium, Trichoderma, Penicilium, Chaetomium, Stachybotrys, Alternaria* and *Cladosporium*, as well as their teleomorphic genera. Records of compounds reported from studies where the fungal culture was considered incorrectly identified were corrected, before addition to the compound database. When obtained from our own data or the literature, the full UV/VIS spectrum was linked to the record.

Many compounds were further registered to sub-genus level / species group level based on taxonomic data and chemotaxonomic studies [4-10]. In *Aspergillus* these were: *A. niger* complex; *A. nidulans* complex; and *A. fumigatus* complex. In *Fusarium* these were: Arthrosporiella (*F. incarnatum*); Discolor (*F. graminearum*); Elegans (*F. oxysporum*); Eupionnotes (*F. merismoides*); Gibbosum (*F. equiseti*); Lateritium (*F. lateritium*); Liseola (*F. verticillioides*); Martiella (*F. solani*); Roseum (*F. avenaceum*); and Sporotrichiella (*F. poae*).

From our work on metabolite profiling genera such as *Aspergillus, Fusarium, Penicillium, Alternaria* and *Cladosporium*, approximately 400 unknown compounds were added to the database as “unknowns” and registered via their elemental composition and from which species the compounds were detected.

For each compound the known or suspected major adducts, based on analysis of reference standards, were listed as: $[M+H]^+$, $[M+Na]^+$, $[M+NH_4]^+$, $[M+K]^+$, $[M+H+CH_3CN]^+$, $[M+Na+CH_3CN]^+$, $[M+H-H_2O]^+$, $[M+H-2H_2O]^+$, $[M+H-H_2]^+$(sterols), $[M+H-HCOOH]^+$, $[M+H-CH_3COOH]^+$, $[M+2H]^{2+}$, $[M+Na+H]^{2+}$ or $[M+2Na]^{2+}$ or “No ionization” in ESI^+, and in ESI^-: $[M-H]^-$, $[M-H+HCOOH]^-$, and $[M+Cl]^-$.

Creating search lists for Target Analysis (TA)

A Microsoft Excel application was created so the whole Chemfolder data-base (without structures) could be copied into one of the Excel sheets, and then sorted to include one or more genera, subspecies, known impurities, or all compounds with unknown retention time (RT). These data were transferred to a data search-list for TA containing: RT (if known), elemental composition and charge state of desired adduct, and name of compound.

For labelling of peaks in Bruker DataAnalysis 4.0 (DA) (Bruker Daltonics, Bremen, Germany), compounds that were available as reference standards were labelled “S-x“ in front of the name where x is the reference standard number in our database. Compounds observed in sample blanks, were labelled “Bl-“ in front of the name. Finally, compounds not tentatively identified were labelled as “Unknown”-”producing species”-number in the species, e.g. “Unknown-Aspergillus nidulans No. 3”.

Automated screening of fungal samples

TA 1.2 (Bruker Daltonics, Bremen, Germany), was used to process data-files with the following typical parameters: A) retention time (if known) as ± 1.2 min (broad range), 0.8 min (medium range) and 0.3 min (narrow range); B) SigmaFit; broad 1000 (isotope fit not used), 40 as medium, and 20 as narrow range; and C) mass accuracy of the peak assessed at 4 ppm (broad range), 2.5 ppm (medium range), and 1.5 ppm (narrow range). Area cut off was set to 3000 counts as default, but was often adjusted in case of very concentrated or dilute samples.

The Software DA was used for manual comparison of all the extracted-ion-chromatograms (EIC), generated by TA, to the BPC chromatograms in order to identify non-detected major peaks.

Section 2. Aggressive dereplication (AD) of a *Penicillium melanoconidium* extract detects nearly all known compounds

P. melanoconidium has formerly been reported to produce penitrem A, sclerotigenin, roquefortine C, meleagrin, oxaline, penicillic acid, verrucosidin and xanthomegnin, based on HPLC-DAD [40].

The extract was examined by the AD method searching for a subset of ~1700 *Penicillium* compounds and additional 700 compounds, and was found to produce a large number of secondary metabolites, see the figure (Fig. S5, Tables S1 and S2).

Previously detected metabolites along with additional families of secondary metabolites are listed in the Table S1 and the full search results list can be seen in the Table S2. Twenty five secondary metabolites could be assigned with a high degree of confidence. Chrysogine, 6-oxopiperidine-2-carboxylic acid, and 8-(methoxycarbonyl)-1-hydroxy-9-oxo-9H-xanthene-3-carboxylic acid were detected for the first time in *P. melanoconidium*, but been found in related *Penicillium* species [41;42]. Eight members of the roquefortine biosynthetic family (end products oxalines) were found, and also further confirmed by UV spectra and retention times. Concerning the penitrems, taxonomic and biosynthetic considerations, in connection with polarity and literature data, were used to verify the presence of penitrem A-F. Furthermore the UV spectrum and RT was the same for the authentic standard of penitrem A. Isomeric compounds of penitrem A such as pennigritrem and the acid hydrolysis products thomitrem A [43] could be excluded based on UV spectra different from that of penitrem A or because they were minor compounds (pennigritrem) as compared to the main product penitrem A [44;45]. PF1101A and B had the penitrem A UV spectrum which is different from the shearinine and janthitrems [46] and penitrems molecules were therefore much more likely candidates. Biosynthetic and taxonomic considerations also dictate that it must be the penitrems that are produced by *P. melanoconidium*.

The polyketides penicillic acid and verrucosidins were also found in *P. melanoconidium*. Verrucosidin had the same molecular formula as atranone A ($C_{24}H_{32}O_6$) [12], but the UV spectrum easily verified the right one. The finding of normethylverrucosidin and deoxyverrucosidin [47] also confirms that the verrucosidin

biosynthetic family was produced by *P. melanoconidium,* which is likely as the closely related *P. polonicum* and *P. aurantiogriseum* also produce these [40]. A metabolite with the formula $C_{24}H_{32}O_4$ was annotated as 6-farnesyl-5,7-dihydroxcy-4-methylphthalide. However this metabolite has a mycophenolic acid chromophore, which has never been found in *P. melanoconidium*. The formula could be hypothesized to be a "dideoxyverrucosidin", but this has to be confirmed.

Primary metabolites were few, and included: choline-O-sulfate, linoleic acid, phenylalanine and 1,2-dilininoyl-n-glycero-3-phosphocholine, which could be annotated based on reference standards. In conclusion several new families of compounds were which are highly toxic, especially the verrucosidins, but also chrysogine a compound often detected in cereal infecting fungi, e.g. *Fusarium*. Such information is valuable for future comparative genomics for revealing biosynthetic pathways.

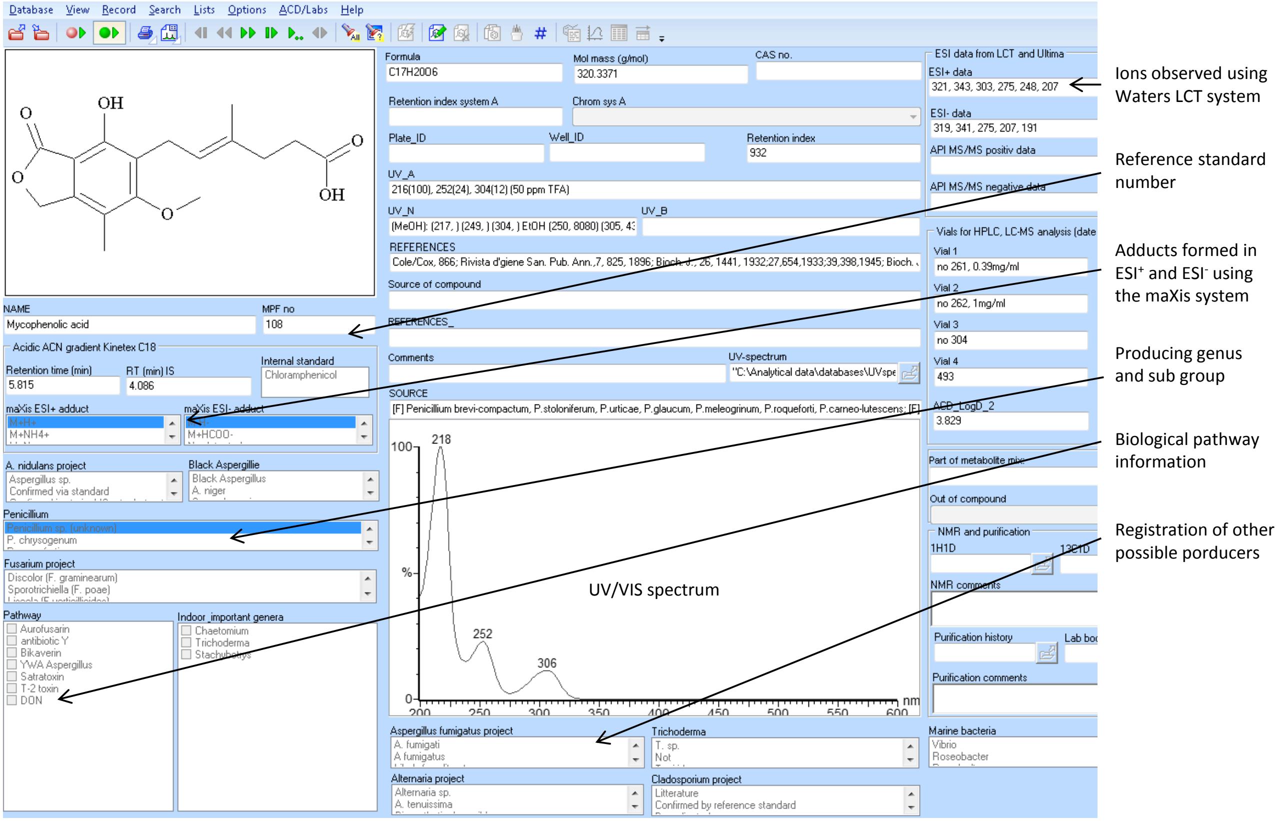

Fig. S1. Compound registration in the compound database

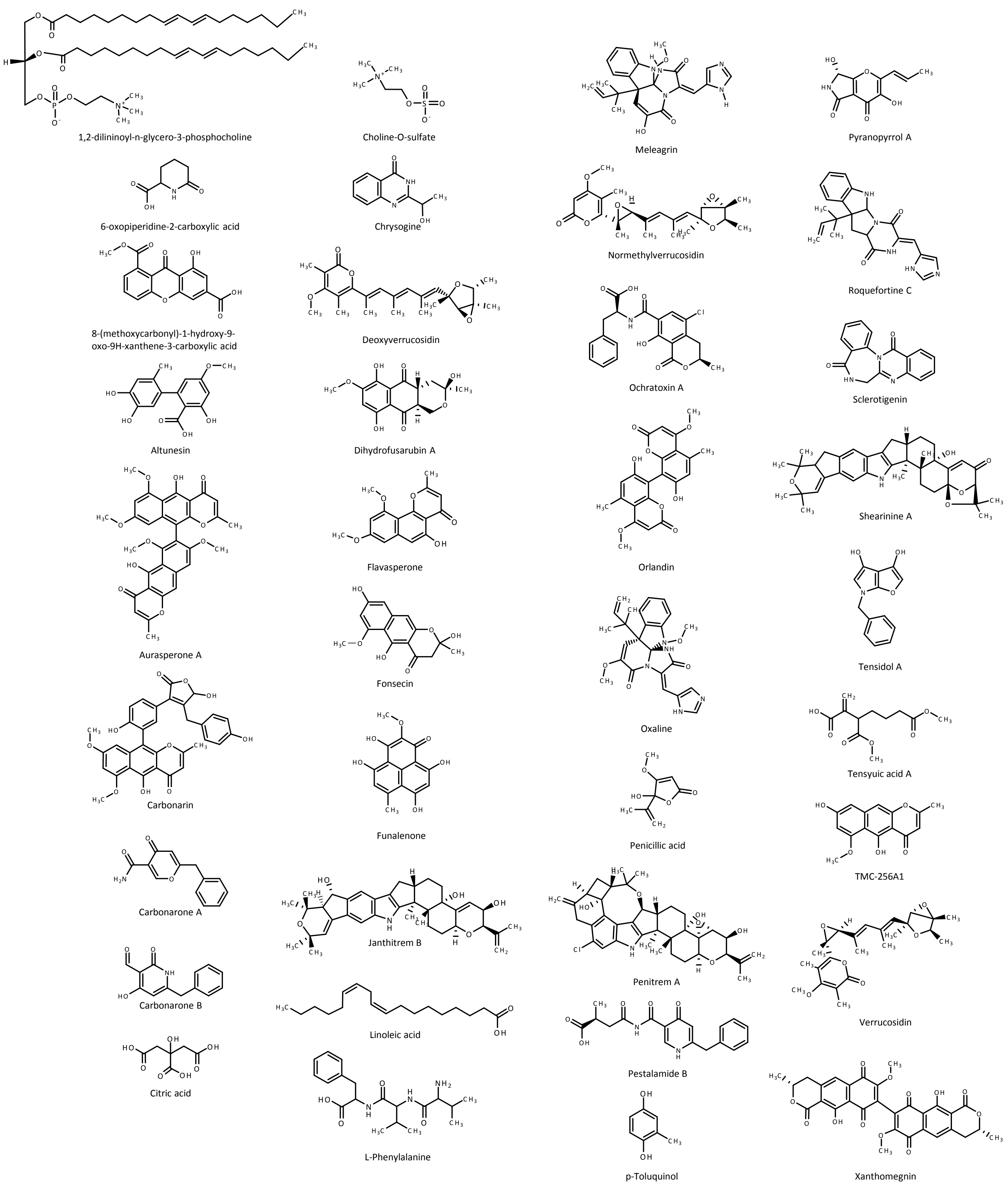

Fig. S2. Chemical structures of compounds mentioned in the text. The structures are shown in alphabetical order in columns from left to right. Only one example for each biosynthetic family is depicted

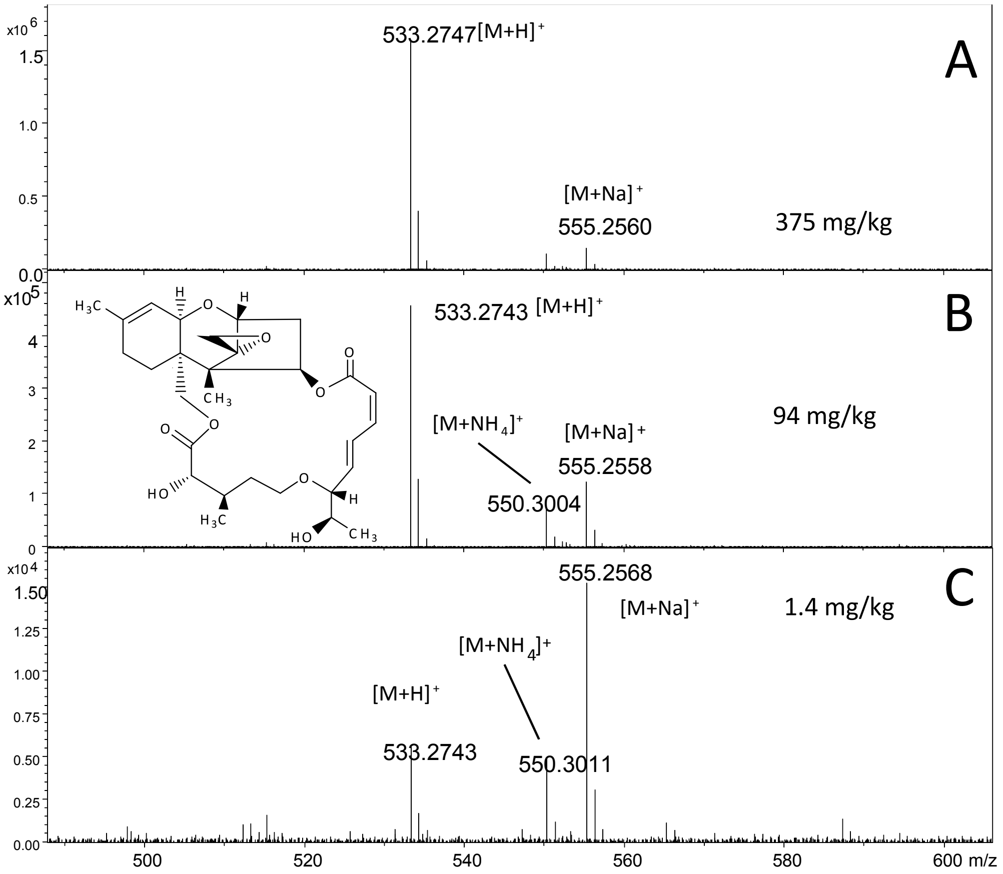

Fig. S3. ESI$^+$ spectrum of roridin A in crude extracts of *Baccharis megapotamica* spiked with (A) 375, (B) 94 and (C) 1.4 mg/kg roridin A

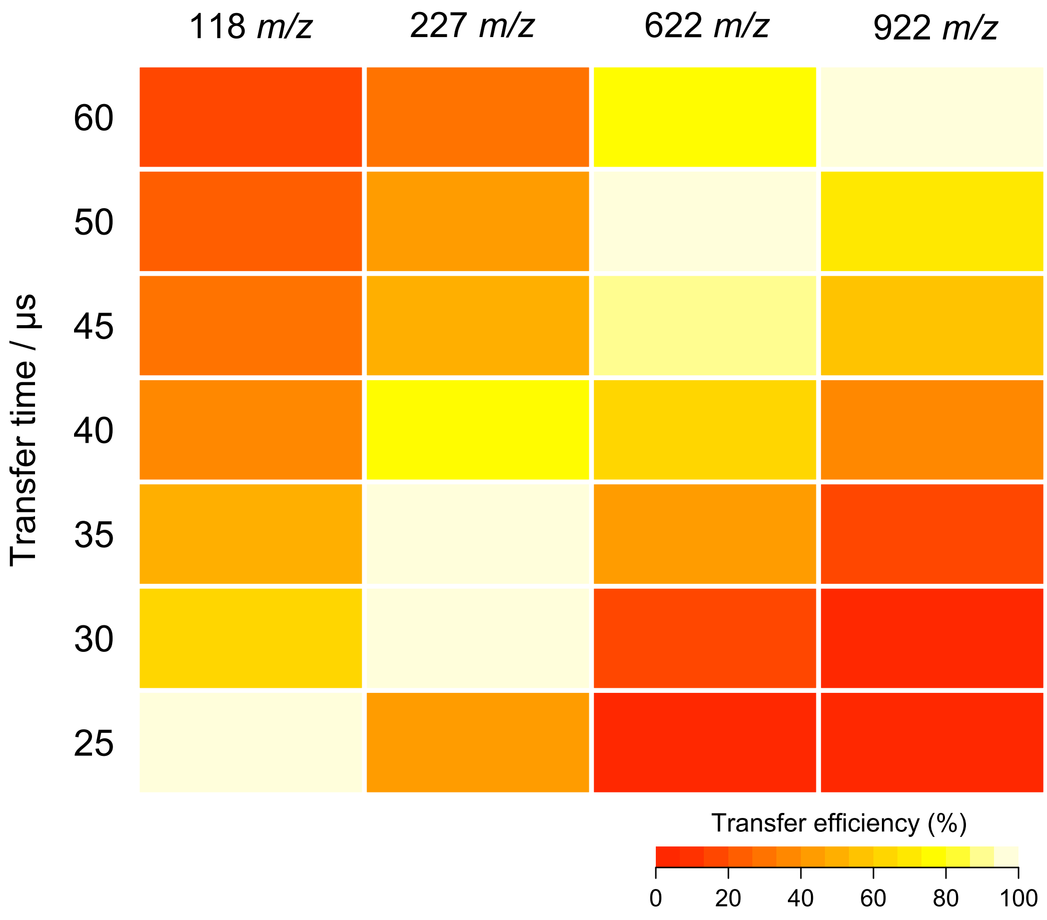

Fig. S4. Transfer efficiency (%) of selected ions from *m/z* 118-922 (relative to maximum)

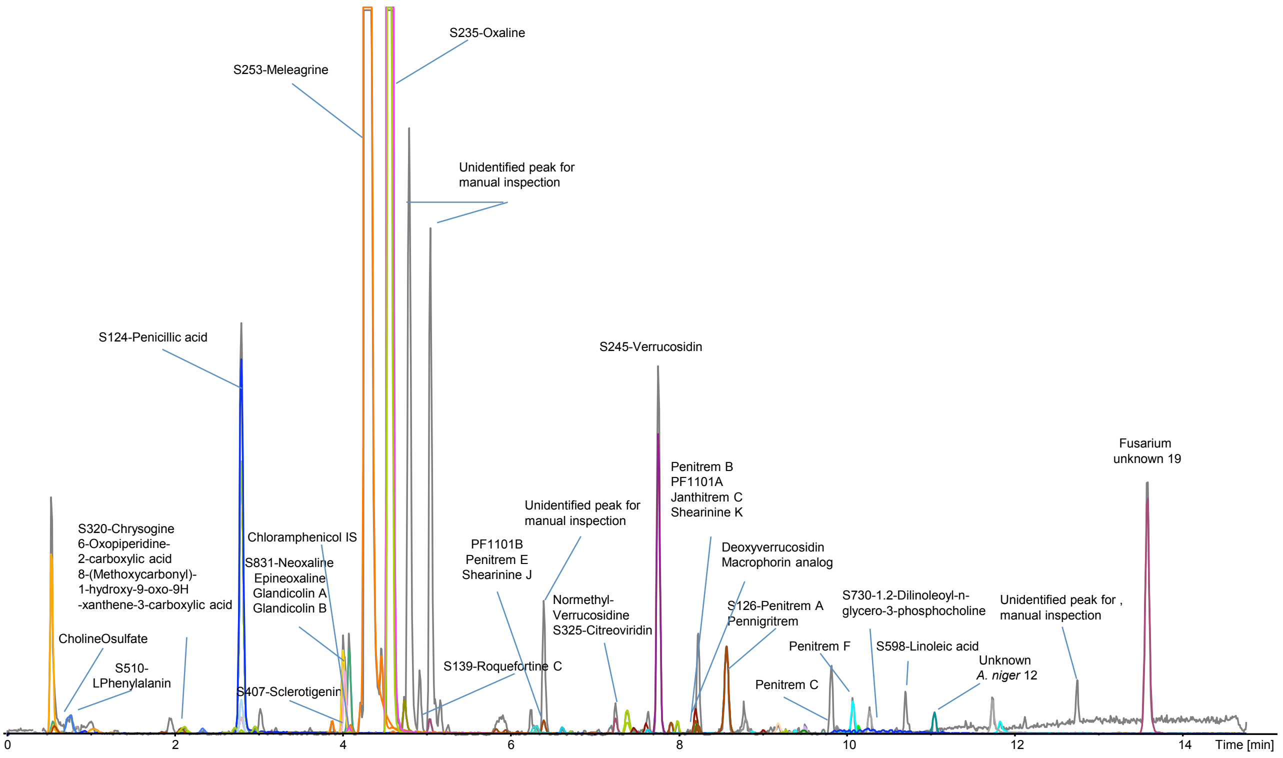

Fig. S5. Analyzed fungal extract from *Penicillium melanoconidium* (IBT 30549) cultivated on CYA media. The chromatogram is overlaid with EICs from detected compounds facilitating easy dereplication. The chromatogram has been scaled to better illustrate the presence of smaller peaks

Table S1. UHPLC-HRMS detection of secondary metabolites produced by *Penicillium melanoconidium* IBT 30549 grown on CYA agar for 7 days at 25°C in darkness

Biosynthetic family	Name of metabolite	Formula	Retention time (min.)
Chrysogines	Chrysogine	$C_{10}H_{10}N_2O_2$	2.337
Sclerotigenins	Sclerotigenin	$C_{16}H_{11}N_3O_2$	3.876
Roquefortines	Roquefortine C	$C_{22}H_{23}N_5O_2$	4.738
	Roquefortine F	$C_{23}H_{25}N_5O_3$	5.038
	E-3-H-Imidazol-4-yl-methylene-6-1H-indole-3-yl-methyl-2,5-piperazinedione	$C_{17}H_{15}N_5O_2$	1.038
	Glandicolin A	$C_{22}H_{21}N_5O_3$	4.092
	Glandicolin B	$C_{22}H_{21}N_5O_4$	4.008
	Meleagrin	$C_{23}H_{23}N_5O_4$	4.291
	Epi-Meleagrin)	$C_{23}H_{23}N_5O_4$	4.456
	Epi-Neoxaline	$C_{23}H_{25}N_5O_4$	4.028
	Oxaline	$C_{24}H_{25}N_5O_4$	4.560
Penitrems	Penitrem A	$C_{37}H_{44}ClNO_6$	8.563
	Penitrem B	$C_{37}H_{45}NO_5$	8.217
	Penitrem C	$C_{37}H_{44}ClNO_4$	9.876
	Penitrem D	$C_{37}H_{45}NO_4$	7.980
	Penitrem E	$C_{37}H_{45}NO_6$	6.613
	Penitrem F	$C_{37}H_{44}ClNO_5$	10.065
	Thomitrem A	$C_{37}H_{44}ClNO_6$	8.226
	PF1101A	$C_{37}H_{47}NO_4$	6.391
	(PF1101A-isomer)	$C_{37}H_{47}NO_4$	8.194
	PF1101B	$C_{37}H_{47}NO_6$	6.309
Penicillic acids	Penicillic acid	$C_8H_{10}O_4$	2.795
Verrucosidins [61]	Verrucosidin	$C_{24}H_{32}O_6$	7.752
	Normethylverrucosidin	$C_{23}H_{30}O_6$	7.245
	Deoxyverrucosidin	$C_{24}H_{32}O_5$	8.197
	Dideoxyverrucosidin	$C_{24}H_{32}O_4$	9.494
Unknown	8-(Methoxycarbonyl)-1-hydroxy-9-oxo-9H-xanthene-3-carboxylic acid	$C_{16}H_{10}O_7$	2.118
Unknown	Toluquinol	$C_7H_8O_2$	2.794
Primary metabolites	Cholin-O-sulfate	$C_5H_{13}NO_4S$	0.561
	Phenylalanine	$C_9H_{11}NO_2$	0.757
	1,2-dilininoyl-n-glycero-3-phosphocholine	$C_{44}H_{80}NO_8P$	10.237
	Linoleic acid	$C_{18}H_{32}O_2$	10.265

Table S2. Table S2 – AD of extract of *P. melanoconidium* grown on CYA agar (crude results)

Peak	Class	Comment	Compound Name	Mol.Formula	Err ppm	mSigma	Area	RT meassured	RT expected
A	+++		Unknown A nidulans no 37 Diana	C6H13NaO6	0.9	9	240479	0.54	0.64
B	+++		BL-UK Cla no 32 possible blank	C7H13NO2	0.4	19	19325	0.558	0.57
C	+		CholineOsulfate	C5H13NO4S1	0.1	25	12403	0.561	0.00
D	+++		BL-UK Cla no 60 possible blank	C10H13N5O4	0.4	32	14268	0.577	0.72
E	+		S510-LPhenylalanin	C9H11NO2	2.4	2	53072	0.757	
E	++		BL-UK Cla no 54 possible blank	C9H11NO2	2.4	2	53072	0.757	0.85
F	+	Detected for the first time in P. melanoconidium	6Oxopiperidine2carboxylic acid	C6H9NO3	1.2	12	16102	0.834	
G	+		E31HImidazol4ylmethylen61Hindol3ylmethyl2.5piperazindiol	C17H15N5O2	1	50	17266	1.038	
G	+		E31HImidazol4ylmethylene61Hindole3ylmethyl2.5piperazinediol	C17H15N5O2	1	50	17266	1.038	
H	+++		BL-UK Cla no 95 possible blank	C7H14N2O3	1.6	17	11833	2.084	2.10
H	+++		BL-UK Cla no 94 possible blank	C7H14N2O3	1.6	17	11833	2.084	1.91
I	+	Detected for the first time in P. melanoconidium	8Methoxycarbonyl1hydroxy9oxo9Hxanthene3carboxylic acid	C16H10O7	1.7	32	19042	2.118	
J	+++	Detected for the first time in *P. melanoconidium*	S320-Chrysogine	C10H10N2O2	1.5	28	10415	2.337	2.56
K	+	No, confused with toloquinol	2.3Dihydroxy toluene	C7H8O2	2.3	3	32022	2.794	
K	+		S297-Hydroquinone. methyl 6CI.8CI	C7H8O2	2.3	3	32022	2.794	
K	+		2Acetyl5methylfuran	C7H8O2	2.3	3	32022	2.794	
K	+		S502-3.5dihydrotoluen	C7H8O2	2.3	3	32022	2.794	
L	+		S124-Penicillic acid	C8H10O4	2.2	15	660761	2.795	
M	+		8betaHydroxy7oxocurvularin	C16H18O7	1.2	11	51254	2.796	
M	+		11aHydroxy12oxocurvularin	C16H18O7	1.2	11	51254	2.796	
M	+		S103-6Methylsalicylic acid	C8H8O3	0.6	15	484020	2.796	
M	+		S601-3hydroxy4methylbenzoic acid	C8H8O3	0.6	15	484020	2.796	
M	+		S621-2hydroxy3methoxybenzaldehyde	C8H8O3	0.6	15	484020	2.796	
M	+		S620-3hydroxy4methoxybenzaldehyde	C8H8O3	0.6	15	484020	2.796	
M	+		S570-pHydroxybenzoic acid methyl ester	C8H8O3	0.6	15	484020	2.796	
M	+		S616-12.6dihydroxyphenylethanone	C8H8O3	0.6	15	484020	2.796	
M	+		S499-3Methylsalicylic acid	C8H8O3	0.6	15	484020	2.796	
N	+++		BL-UK Cla no 11 possible blank	C11H18N2O2	0.1	21	12754	2.957	2.85
N	+++		BL-UK Cla no 12 possible blank	C11H18N2O2	0.1	21	12754	2.957	3.09
O	+++		S407-Sclerotigenin	C16H11N3O2	0.4	10	18700	3.876	3.88
P	+		Sorbicillactone B	C21H25NO8	2.2	6	124492	4.008	
P	+++		Glandicolin B	C22H21N5O4	0.9	20	123717	4.008	4.01
Q	+++		S831-Neoxaline	C23H25N5O4	0.5	18	86551	4.028	4.28
Q	+		epiNeoxaline	C23H25N5O4	0.5	18	86551	4.028	
R	+++	Internal standard	Chloramphenicol IS	C11H12Cl2N2O5	0.2	26	129956	4.078	4.12
S	+++		Glandicolin A	C22H21N5O3	0.1	4	14081	4.092	4.09
T	+++		S253-Meleagrin	C23H23N5O	1	24	1E+07	4.291	4.29

				4					
U	+++		S253-Meleagrin	C23H23N5O4	0	24	319359	4.456	4.29
V	++		UK Cla no 61	C20H32O11	1.1	54	2092310	4.56	5.33
V	+++		S235-Oxaline	C24H25N5O4	1.3	7	7560329	4.56	4.56
V	+		S470-Oxaline	C24H25N5O4	1.3	7	7560329	4.56	
X	+		S340-PF3	C22H23N5O2	0.4	7	59076	4.738	
X	+++		S139-Roquefortine C	C22H23N5O2	0.4	7	59076	4.738	4.97
X	+		S338-PF1	C22H23N5O2	0.4	7	59076	4.738	
Y	++		Fusarium solani unknown 15	C22H29N1O7	3.2	11	21556	5.038	5.52
Y	+++		Roquefortine F	C23H25N5O3	0	17	21760	5.038	5.04
Z	++		Unknown in A. niger 20	C28H36N4O5	2.4	23	10745	6.273	6.04
AA	+		PF1101B	C37H47NO6	1.6	50	12191	6.309	
AA	+	No, confused with penitrem-like compound	Shearinine J	C37H47NO6	1.6	50	12191	6.309	
AB	+	No, confused with penitrem-like compound	Shearinine K	C37H47NO4	1	19	19716	6.391	
AB	+		PF1101A	C37H47NO4	1	19	19716	6.391	
AB	+	No, confused with penitrem-like compound	Janthitrem C	C37H47NO4	1	19	19716	6.391	
AC	+		Thomitrem E	C37H45NO6	1.9	59	10690	6.613	
AC	+		S387-Penitremone A	C37H45NO6	1.9	59	10690	6.613	
AC	+	No, confused with penitrem-like compound	Shearinine D	C37H45NO6	1.9	59	10690	6.613	
AC	++		Penitrem E	C37H45NO6	1.9	59	10690	6.613	6.61
AD	+++		Normethylverrucosidine	C23H30O6	0.6	12	21959	7.245	7.25
AD	+		S37-Citreoviridin	C23H30O6	0.6	12	21959	7.245	
AD	+		S325-Citreoviridin	C23H30O6	0.6	12	21959	7.245	
AE	+		IsocitreohybridoneB	C29H38O8	2.5	22	44729	7.383	
AE	+		Citreohybridone B	C29H38O8	2.5	22	44729	7.383	
AF	+++		Unknown in A. niger 21	C27H40O8	1.9	29	20444	7.384	7.49
AG	+++		Unknown A carbonarius no 9	C29H41N7O2	0.9	16	14010	7.608	7.69
AH	++	No, confused with verrucosidin	S452-AtranoneA	C24H32O6	0.5	34	439654	7.752	7.23
AH	+++		S245-Verrucosidin	C24H32O6	0.5	34	439654	7.752	7.75
AI	++		Fusarium solani unknown 11	C18H31NaO4	0.4	21	17273	7.904	7.29
AI	++		Fusarium solani unknown 10	C18H31NaO4	0.4	21	17273	7.904	7.16
AJ	+++		Penitrem D	C37H45NO4	1.1	14	19122	7.98	7.98
AK	+	No, confused with penitrem-like compound	Shearinine K	C37H47NO4	1.4	20	18744	8.194	
AK	+		PF1101A	C37H47NO4	1.4	20	18744	8.194	
AK	+	No, confused with penitrem-like compound	Janthitrem C	C37H47NO4	1.4	20	18744	8.194	
AL	+		Macrophorin analog	C24H32O5	1	8	38721	8.197	
AL	+++		Deoxyverrucosidin	C24H32O5	1	8	38721	8.197	8.20
AM	+++		Penitrem B	C37H45NO5	1.9	36	15171	8.217	8.22
AM	+		Shearinine F	C37H45NO5	1.9	36	15171	8.217	
AM	+		Penitremone C	C37H45NO5	1.9	36	15171	8.217	
AM	+		ShearinineA	C37H45NO5	1.9	36	15171	8.217	

AN	+	No, confused with penitrem A	Pennigritrem	C37H44ClN1 O6	2	26	12720	8.226	
AN	+	No, confused with penitrem A	Thomitrem A	C37H44ClN1 O6	2	26	12720	8.226	
AN	++	No, confused with penitrem A	S126-Penitrem A	C37H44ClN1 O6	2	26	12720	8.226	8.56
AO	+		Pennigritrem	C37H44ClN1 O6	2.1	42	172731	8.563	
AO	+		Thomitrem A	C37H44ClN1 O6	2.1	42	172731	8.563	
AO	++		S126-Penitrem A	C37H44ClN1 O6	2.1	42	172731	8.563	8.56
AP	++		Unknown in A. niger 18	C16H21NaO4	3.4	14	31776	8.794	8.85
AQ	+++		Unknown in A. niger 24	C28H42	1.8	23	18486	9.179	8.93
AR	+		6-Farnesyl-5,7-dihydroxy-4-methylphthalide	C24H32O4	2.9	49	10051	9.494	
AS	+++		Penitrem C	C37H44ClNO 4	0.1	33	11419	9.876	9.88
AT	+++		Penitrem F	C37H44ClNO 5	1.6	25	53231	10.07	10.07
AU	++		Unknown A nidulans no 36 Diana	C19H37NaO4	1.3	37	12976	10.13	9.67
AV	+++		S730-1,2-Dilinoleoyl-sn-glycero-3-phosphocholine	C44H80NO8 P	0.2	17	24565	10.24	10.24
AX	+++		S598-Linoleic acid	C18H32O2	1.5	7	38041	10.27	10.17
AY	+++		Unknown in A. niger 12	C21H41NaO4	0.5	9	35385	11.04	11.04
AZ	+++		Fusarium solani unknown 2	C24H37NaO4	0.1	10	62599	11.73	11.67
AA A	+++		BL-UK Cla no 83 possible blank	C22H43NO	0.5	10	28484	11.82	11.84
AA B	++		Fusarium unknown 19	C27H21N2Na O9	1.5	82	470182	13.57	13.49

mSigma: Fit of isotope pattern, see text for more. RT Retention time (min).

References

1. Nielsen KF, Månsson M, Rank C, Frisvad JC, Larsen TO (2011) Dereplication of microbial natural products by LC-DAD-TOFMS. J Nat Prod 74:2338-2348

2. Rank C, Klejnstrup ML, Petersen LM, Kildgaard S, Frisvad JC, Godtfredsen CH, Larsen TO (2012) Comparative chemistry of *Aspergillus oryzae* (RIB40) and *A. flavus* (NRRL 3357). Metabolites 2:39-56

3. Månsson M, Phipps RK, Gram L, Munro MH, Larsen TO, Nielsen KF (2010) Explorative Solid-Phase Extraction (E-SPE) for Accelerated Microbial Natural Product Discovery, Dereplication, and Purification. J Nat Prod 73:1126-1132

4. Nielsen KF, Mogensen JM, Johansen M, Larsen TO, Frisvad JC (2009) Review of secondary metabolites and mycotoxins from the *Aspergillus niger* group. Anal Bioanal Chem 395:1225-1242

5. Frisvad JC, Rank C, Nielsen KF, Larsen TO (2009) Metabolomics of *Aspergillus fumigatus*. Med Mycol 47:S71

6. Rank C, Nielsen KF, Larsen TO, Varga J, Samson RA, Frisvad JC (2011) Distribution of sterigmatocystin in filamentous fungi. Fungal Biology 115:406-420

7. Frisvad JC, Andersen B, Thrane U (2008) The use of secondary metabolite profiling in chemotaxonomy of filamentous fungi. Mycol Res 112:231-240

8. Andersen B, Sørensen JL, Nielsen KF, van den Ende B, de Hoog S (2009) A polyphasic approach to the taxonomy of the *Alternaria infectoria* species-group. Fungal Genet Biol 46:642-656

9. Andersen B, Dongo A, Pryor BM (2008) Secondary metabolite profiling of Alternaria dauci, A. porri, A. solani, and A. tomatophila. Mycol Res 112:241-250

10. Frisvad JC, Smedsgaard J, Larsen TO, Samson RA (2004) Mycotoxins, drugs and other extrolites produced by species in *Penicillium* subgenus *Penicillium*. Stud Mycol 49:201-241
